# Relationship Between National Residency Matching Program (NRMP) Rank Order and Otolaryngology Residency Performance

**DOI:** 10.1002/oto2.127

**Published:** 2024-04-03

**Authors:** Uche C. Ezeh, Mario A. Svirsky, Max M. April

**Affiliations:** ^1^ Division of Pediatric Otolaryngology, Department of Otolaryngology–Head and Neck Surgery New York University School of Medicine New York City New York USA; ^2^ Department of Neuroscience and Physiology New York University School of Medicine New York City New York USA

**Keywords:** education, otolaryngology, resident performance, resident ranking

## Abstract

**Objective:**

The process of resident recruitment is costly, and our surgical residency program expends significant time on the resident selection process while balancing general duties and responsibilities. The aim of our study was to explore the relationship between otolaryngology–head and surgery (OHNS) residents' National Residency Matching Program (NRMP) rank‐list position at our institution and their subsequent residency performance.

**Study Design:**

Retrospective cohort study.

**Setting:**

Single site institution.

**Methods:**

We retrospectively reviewed 7 consecutive resident classes (2011‐2017) at a single tertiary OHNS residency program. We reviewed each resident's absolute rank order in the NRMP matches. Measures of residency performance included overall faculty evaluation during postgraduate year 5 (PGY5), annual in‐service examination scores (scaled score), and the number of manuscripts published in peer‐reviewed journals. Correlations between NRMP rank order and subsequent residency performance were assessed using Spearman's rho correlation coefficients (*ρ*).

**Results:**

Twenty‐eight residents entered residency training between 2011 and 2017. The average rank position of the trainees during this study was 9.7 (range: 1‐22). We found no significant correlation between rank order and faculty evaluation during PGY5 (*ρ* = 0.097, *P* = .625) or number of publications (*ρ* = −0.256, *P* = .189). Additionally, when assessing the association between rank order and annual Otolaryngology Training Examination‐scaled scores, no statistically significant relationship was found between the 2 (*P* > .05).

**Conclusion:**

Our results showed that there were no significant correlations between OHNS rank order and various measures of success in residency training, which aligns with existing literature. Further investigation of this relationship should be conducted to ensure the applicability of our findings.

Otolaryngology–head and neck surgery (OHNS) is regarded as one of the most competitive medical specialties in the United States. From 2021 to 2022, 574 medical school graduates applied for 361 OHNS residency seats, resulting in a 63% match rate, which is a 10% decline from 2011 to 2012.^1^, ^2^ Like other surgical specialties, OHNS training programs are challenged with the task of screening many high‐achieving candidates for a smaller cohort of students for a limited number of seats. The resident selection process is important for programs to predict a candidate's future success as a resident and an otolaryngologist.

During the evaluation process, residency selection committees consider various factors for interview selection and National Residency Matching Program (NRMP) rank list generation: United States Medical Licensing Examination Step 1 and 2 scores, Medical Student Performance Evaluation, medical school transcripts, letters of recommendations, Alpha Omega Alpha status, research production, and interview performance.^3^, ^4^ Calhoun and colleagues have found several of these factors to be predictive of future OHNS residency performance.^4^, ^5^, ^6^

Investigations into whether the OHNS applicant rank order is associated with future success are very limited. Only 1 prior study has evaluated this correlation among OHNS residents. In 2011, Bent et al^7^ retrospectively analyzed a cohort of residents in a single OHNS program (2001‐2008) by assessing whether there was a correlation between their rank order and subsequent residency performance—faculty and peer‐resident evaluations during postgraduate year 5 (PGY5), chief resident selection, graduating resident teaching award selection, and in‐service examinations. That study found that the rank order did not correlate with any of these performance parameters. These findings align with the results of most published studies investigating the correlation or predictive value of NRMP rank order on subsequent residency performance across various specialities including radiology, internal medicine, and emergency medicine.^8^, ^9^, ^10^, ^11^, ^12^

Considering the recent trends in OHNS competitiveness and the paucity of literature examining NRMP rank correlations since the study by Bent et al, it is important to reexamine this relationship. We sought to explore whether NRMP rank order within a large academic OHNS program correlated with residents’ performance.

## Methods

All residents who were trained in the OHNS residency program at New York University Langone Health (NYULH) between 2011 and 2017 were retrospectively reviewed. This specific timeframe was chosen due to the stability in leadership during that period. We identified 28 residents that met the inclusion criteria. There was no resident attrition during the study period. We acquired information about each resident's individual NRMP rank number, and measured residency success using the following outcomes: overall faculty evaluation score during PGY5, annual American Board of Otolaryngology Training Examination (OTE) score, and number of peer‐reviewed publications produced during residency plus 1 year after graduation. Written qualifying examination reports (pass/fail) were not included in the analysis because all residents passed their first attempt. Additionally, peer resident evaluations were inconsistently reported for each trainee and were therefore not included in this study. This project was granted approval by the NYULH Institutional Review Board committee (protocol: i23‐00187). The requirement of individual consent was waived due to the retrospective nature of the study.

### NRMP Rank Order

At the beginning of each application cycle, 5 senior OHNS faculty members evaluated the records of applicants, and each faculty member selected 10 qualified applicants plus 1 alternative for interview consideration. No rank scores were assigned during the screening. Interviews were then offered to 42 to 45 candidates and scheduled over the course of 2 days. There are 5 interview rooms with 2 attendings, at least 1 research faculty member, and 1 PGY4 resident. The sixth room is an interview with only the chairman. The week after the interviews, each interviewer submits their respective rank‐order list. These lists were compiled by an administrator and reordered from the most desirable to the least desirable. This was then sent to the committee for discussion and final adjustments before submission to the NRMP.

### Faculty Evaluations

Each year, residents undergo multiple subjective evaluations of their clinical, operative, and interpersonal skills. Faculty evaluations were used to assess clinical and surgical competencies. At the conclusion of every rotation, faculty rated residents on a 5‐point scale (1 = unsatisfactory; 5 = excellent) for each of the 6 Accreditation Council for Graduate Medical Education (ACGME) general competencies: patient care, medical knowledge, systems‐based practice, problem‐based learning, professionalism, and interpersonal communication skills.

### Otolaryngology Training Exam (OTE) Scores

Every March, residents are expected to sit for the annual OTE to evaluate their competencies across several disciplines including allergy, head and neck, laryngology, otology, pediatrics, facial plastic and reconstructive, rhinology, and sleep.

Prior to 2011, the in‐service scores were reported as percentiles. However, since then, the American Board of Otolaryngology Board of Directors changed the score reporting to include a scaled score and stanines (“standard score of nine categories”). For this study, we analyzed the “scaled scores” given that they are associated with raw scores.

### Research Productivity

In contrast to the Bent et al study, we also examined whether there was a correlation between rank order and the number of publications produced during residency training. The PubMed research database was searched for peer‐reviewed publications by each resident. Each search involved first and last names. If necessary, a middle initial was included. We imposed a time restriction of 5 years on the search, coinciding with the duration of each resident's training period. Furthermore, we included 1 additional postgraduate year to factor for publication lag time. We verified that each article was published during the residency period by confirming that the resident and coauthors or senior authors were affiliated with New York University (NYU).

### Statistical Analysis

The associations between the rank order and residency performance metrics were evaluated using Spearman's rho correlation coefficients. Spearman *R* values of <.2 of .2 to .39 are conventionally considered a weak correlation, *R* values of .40 to .59 were considered a moderate correlation, *R* values of .6 to .79 are considered a strong correlation and *R* > .8 are considered very strong correlations. All statistical analyses were performed using SPSS 28.0 (IBM). Statistical significance was set at *P* < .05.

## Results

**Figure 1:**
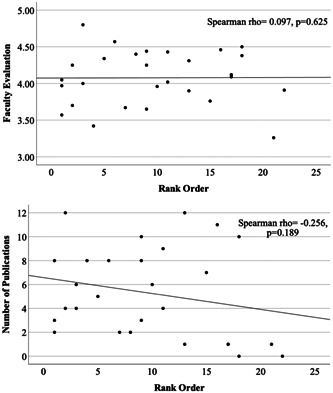
Spearman's rank correlation scatter plot of faculty evaluation scores and number of manuscripts produced against rank order.

**Figure 2:**
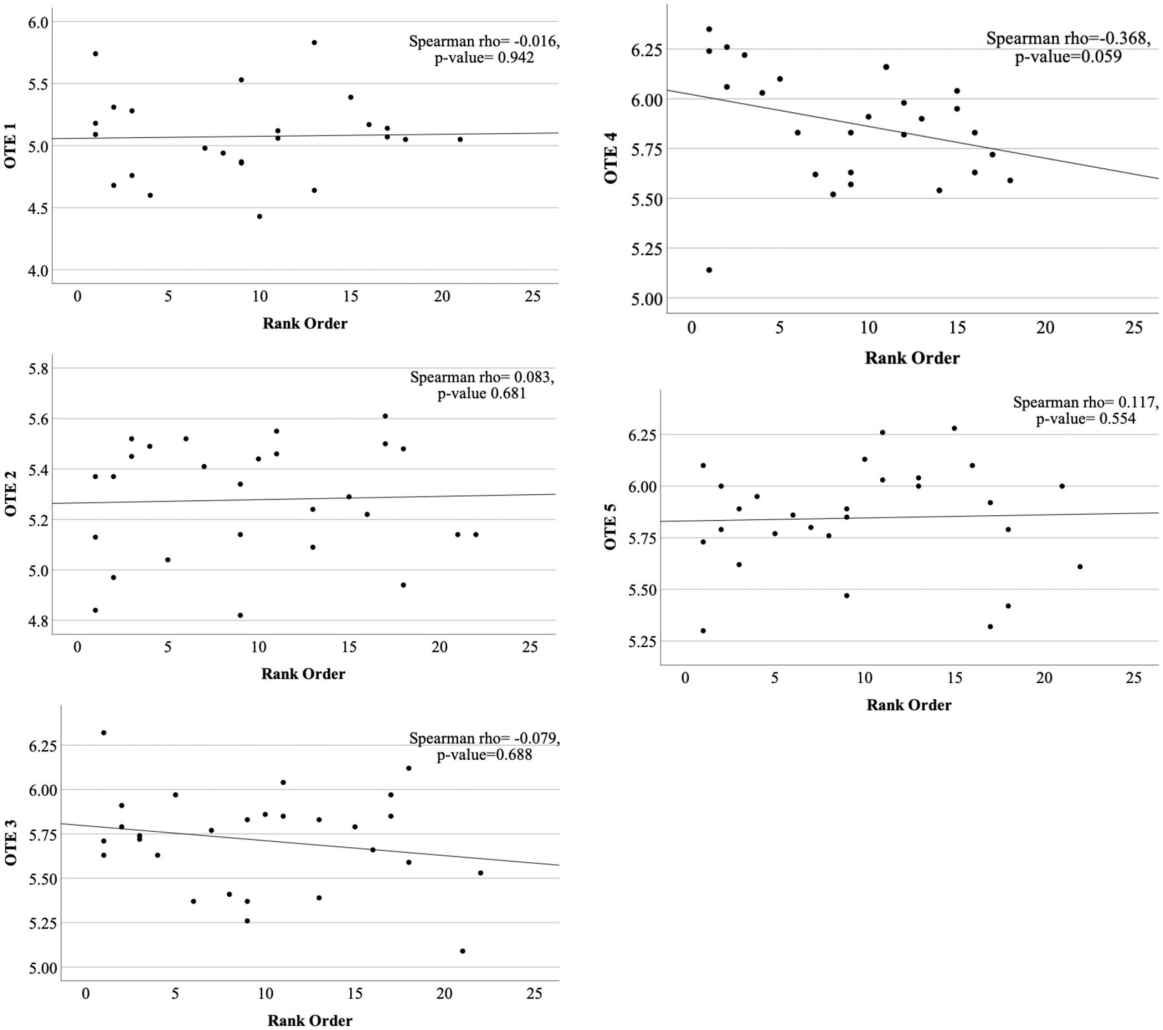
Spearman's rank correlation scatter plot of annual otolaryngology training exam (OTE) scaled scores against rank order.

Twenty‐eight OHNS residents from 2011 to 2017 were included in this study. The mean resident NRMP rank number was 9.7 (range: 1‐22). We found no significant correlation between rank order and faculty evaluation during PGY5 (*ρ* = 0.097, *P* = .625) or number of publications (*ρ* = −0.256, *P* = .189) (Figure 1). Additionally, when assessing the association between rank order and annual OTE‐scaled scores, we failed to find any statistically significant relationship between the 2 (*P* > .05) (Figure 2).

## Discussion

This study aimed to assess the extent to which our current residency selection system is predictive of resident performance by examining the association between the NRMP final rank order and OHNS residency success. We measured residency performance using faculty evaluation scores during the senior year, annual OTE examination scores, and the number of publications during residency. We initially hypothesized that higher‐ranked applicants who matched our OHNS residency program were more likely to perform better during training than residents who were assigned a lower rank. However, this hypothesis was not supported.

Instead, our findings were consistent with those of Bent et al. Their study also found that the match‐rank order did not significantly correlate with the chief resident selection or reception of the resident teaching award. Our OHNS residency program does not assign a teaching award and all PGY5 residents are assigned the title “chief,” hence our study could not assess these variables.

While there is a paucity of literature in the field of OHNS that evaluates this correlation, our present findings may not be surprising. Many investigators have demonstrated that there is little to no correlation or association between NRMP rank order and performance during residency,^8^, ^9^, ^10^, ^11^, ^12^, ^13^ whereas few have found significant relationships between the 2.^14^, ^15^, ^16^

One reason for the poor correlation between the NRMP rank order and residency performance could be the influence of subjective opinions during the rank committee review. After completing the interviews, most programs have a committee composed of the program director, attendings, and residents, who all discuss interviewee applications and then assign a numerical rank number for each candidate applicant that is submitted to the NRMP match. There are factors outside of the medical student ERAS application that may influence where an applicant falls on a rank list, including family situation, personal ties to the program, and fit with program culture and values. Interestingly, studies in both plastics and general surgery have shown that the unadjusted rank list position was more predictive of residency performance than the adjusted rank list position,^12^, ^13^ which suggests that certain factors after interview selection may result in rank position being a poor correlator of residency performance.

In our study, we may have observed no significant correlation due to data clustering. Like other OHNS programs, NYU matches 4 applicants per cycle. During the study period of 7 years, the highest‐ranked candidate was 1 and the lowest‐ranked candidate was 22. One year, the program successfully matched applicants ranked 1 to 4 and another year matched applicants ranked 9, 10, 11, and 13. When assessing PGY4 and 5 in‐service scores, the range of scaled scores was very small. For instance, during PGY4, the scores ranged between 5.14 and 6.35; during PGY5, they ranged between 5.3 and 6.28. As with any negative result, the possibility remains that a future, larger study may find statistically significant relationships.

Few studies have found strong associations between match‐rank order and subsequent residency performance.^14^, ^15^, ^16^ One study on OBGYN residents found a strong association between rank order percentile and PGY1 clinical performance evaluation scores, which was measured using a scale to rate their ACGME core competencies (*r* = .60, *P* < .001).^14^ However, these findings are not surprising, considering that the study assessed clinical performance during PGY1, which is only 1 year after being matched. Sklar and Tandberg^16^ studied 20 emergency medicine residents and showed that residents perceived by faculty evaluators as being “stronger” were ranked higher on the NRMP rank list. Grewal et al^15^ conducted a study of 29 urology residents who graduated between 2000 and 2009. They measured residency performance by assessing faculty evaluation and in‐service scores. Residents were evaluated by faculty biannually based on a Likert scale and categorized by the investigators as “excellent” or “average and needs improvement.” They stratified the mean in‐service scores over the course of residency as <50th percentile or >50th percentile. Their study found that “excellent” residents and those with >50th percentile on in‐service exams were ranked higher by the program during the NRMP match.

In comparison to most prior studies, we included resident research as a measure of residency performance and studied its relationship with the final rank order. Our study found no significant association between the rank order and publication count. The explanation for this finding is not clear, but it must be noted that there may be variability in these results considering some residents' lack of interest in an academic career, and publications are not the only measure of research productivity (ie, abstracts, poster presentations, and oral presentations). Future studies may evaluate this association because many OHNS residency programs strive to train residents for careers in academic medicine. As of today, many OHNS residency programs have protected research blocks for trainees to complete scholarly projects during their postgraduate years.

This retrospective review has several limitations. First, it assessed a small cohort of residents at a single institution, which limits the generalizability of our data. It is possible that the correlation between an applicant's rank number in terms of future performance as a resident may be stronger in larger programs. However, small sample sizes are a limitation commonly noted in similar studies on this topic.

Furthermore, it should also be noted that, compared to prior studies on NRMP rank order and residency success, OHNS matches a smaller cohort of residents per year compared to internal medicine, pediatrics, emergency medicine, and radiology. This discrepancy raises questions about whether future studies on OHNS residency rank order would benefit from pooled data across several institutions, as this would enhance the external validity of our findings. However, this would be challenging, considering that residency selection processes and criteria are not uniform across different programs.

In addition to these limitations, there are 2 primary challenges when comparing rank orders across different years. First, the meaning of a specific rank list position can vary each year depending on the pool of available and interested applicants. Consequently, comparing the top‐ranked applicants from 1 year to those the following year may raise concerns about the validity and reliability of such a comparison. Also, NRMP rank list order reflects the program's perspective regarding the fit of an applicant to their specific program. The notion of “fit” is highly contextual and may fluctuate within a program over time, introducing inherent variability when attempting to compare NRMP rankings across multiple years.

Regarding research productivity, we defined this as the number of manuscripts published during residency; other additional measures, such as the number of abstracts, posters, and oral presentations completed during residency training, would have been helpful to include to capture a resident's complete scholarly research profile. Additionally, we limited our search to only the PubMed database, which may have underestimated a resident's overall publication count during residency.

Improving our study could involve incorporating additional measures to assess residency performance. The current proxies utilized at our institution for evaluating performance may not be the most reliable indicators of success in OHNS residency. However, a previous systemic review highlighted the scarcity of measures available for evaluating a resident's performance, primarily relying on faculty assessment, in‐training exams, board exams, chief residency selection, and research productivity.^17^

Our findings may lead some to the interpretation that the rank list order might have limited predictive utility when it comes to the future success of OHNS trainees. However, an alternative perspective could be that the resources and training offered provided by our OHNS program may have allowed residents to grow and develop throughout their 5‐year tenure, enabling them to exceed initial ranking expectations.

## Conclusion

The process of resident recruitment is costly, and our OHNS residency program expends significant time on the resident selection process while balancing general duties and responsibilities. Our hypothesis was that our institutions' rank‐list order would positively correlate with residency performance. This data refutes that hypothesis and confirms the previous study by Bent et al that there were no significant correlations between OHNS rank order and various measures of success in residency training.

## Author Contributions

**Uche C. Ezeh**, writing, analysis, figures, editing; **Mario A. Svirsky**, analysis, editing; **Max M. April**, conception, methods, editing.

## Disclosures

### Competing interests

All authors declare no commercial conflicts of interest.

### Funding source

The authors have no funding or financial relationships to disclose with respect to the research presented in this article.
